# Productive Aging in India: Pre-Retirement Perceptions, Priorities, and Gendered Perspectives

**DOI:** 10.1093/geroni/igaa057.370

**Published:** 2020-12-16

**Authors:** Reema Sen, Eva Kahana

**Affiliations:** 1 case western reserve university, Cleveland, Ohio, United States; 2 Case Western Reserve University, Cleveland, Ohio, United States

## Abstract

This study explores the perceptions of urban, middle class, white collar employees in India approaching retirement, to unpack ‘productive aging’ through a specific sociocultural lens. India, is aging fast with limited social security. Projected growth in the 60+ age group is 326% (2000 -2050) and 700% for the 80+. Prior studies largely focused on rural and blue collar adults. This study involves a sample of 76 people’s (41 men, 35 women of relatively high SES) attitudes to aging. Key findings show, self-perceived health status was different between men and women (ordinal regression analysis showed p value 0.034 at 95% CI). 51.22% men rated their health excellent/very good compared with 28.57% women. 12.2% men rated their health fair/poor compared with 25.72% women. Despite this, results of men and women’s perceptions of aging were remarkably similar though living in a country with entrenched gender inequality. Cultural influence was apparent from the gendered difference (p value 0.036, 95% CI) in response to the question “our society frowns upon paid work after retirement” (20% women agreed compared with 4.77% men). Interestingly, despite social constraints 68.58% women agreed that they prefer a paid alternate career after retirement compared with 53.66% men. Another difference (p value 0.006, 95% CI) on the question “I need to be gainfully occupied for my own personal satisfaction” had 100% women reporting they agree or strongly agree as compared with 85.37% men. Findings will be discussed in the context of gender and changing family structures among adults in late middle age.

